# Using Machine Learning on V2X Communications Data for VRU Collision Prediction

**DOI:** 10.3390/s23031260

**Published:** 2023-01-22

**Authors:** Bruno Ribeiro, Maria João Nicolau, Alexandre Santos

**Affiliations:** 1 Department of Informatics, University of Minho, 4710-057 Braga, Portugal; 2 Department of Information Systems, University of Minho, 4804-533 Guimarães, Portugal

**Keywords:** vehicular communications, vulnerable road users, collision prediction, machine learning

## Abstract

Intelligent Transportation Systems (ITSs) are systems that aim to provide innovative services for road users in order to improve traffic efficiency, mobility and safety. This aspect of safety is of utmost importance for Vulnerable Road Users (VRUs), as these users are typically more exposed to dangerous situations, and their vehicles also possess poorer safety mechanisms when in comparison to regular vehicles on the road. Implementing automatic safety solutions for VRU vehicles is challenging since they have high agility and it can be difficult to anticipate their behavior. However, if equipped with communication capabilities, the generated Vehicle-to-Anything (V2X) data can be leveraged by Machine Learning (ML) mechanisms in order to implement such automatic systems. This work proposes a VRU (motorcyclist) collision prediction system, utilizing stacked unidirectional Long Short-Term Memorys (LSTMs) on top of communication data that is generated using the VEINS simulation framework (coupling the Simulation of Urban MObility (SUMO) and Network Simulator 3 (ns-3) tools). The proposed system performed well in two different scenarios: in Scenario A, it predicted 96% of the collisions, averaging 4.53 s for Average Prediction Time (s) (APT) and with a Correct Decision Percentage (CDP) of 41% and 78 False Positives (FPs); in Scenario B, it predicted 95% of the collisions, with a 4.44 s APT, while the CDP was 43% with 68 FPs. The results show the effectiveness of the approach: using ML methods on V2X data allowed the prediction of most of the simulated accidents. Nonetheless, the presence of a relatively high number of FPs does not allow for the usage of *automatic* safety features (e.g., emergency breaking in the passenger vehicles); thus, collision avoidance must be achieved *manually* by the drivers.

## 1. Introduction

In Intelligent Transportation Systems (ITSs), road users can utilize communication technologies to disseminate important information that allows the deployment of applications that can make driving more efficient (e.g., in terms of mobility or safety). Taking safety as an example, these systems can range from basic solutions, such as the broadcast of hazardous location warnings, to more complex ones, e.g., automatic collision prediction.

This aspect of safety is particularly important for Vulnerable Road Users (VRUs), since these entities are usually more exposed to danger and casualties/fatalities, partly due to the fact that their vehicles possess poorer safety mechanisms when in comparison to regular vehicles. As defined by the European Commission [1], VRUs consist of pedestrians, cyclists, motorcyclists and persons with disabilities or reduced mobility and orientation.

Safety systems for VRU vehicles (and in ITSs in general) tend to mostly utilize data that come from sensors or similar advanced sensing systems (e.g., LIDAR and RADAR). However, these systems have performance issues when their line of sight is partial or even non-existent. This issue is aggravated when considering VRU vehicles, due to their high mobility and smaller sizes, which makes their detection challenging to achieve. For instance, a motorcycle that is traveling on a road may not be detected by the sensing system if a large bus is parked right in front of it (the VRU is in a *blind spot*). Thus, the implementation of safety systems that are able to work in non-line-of-sight situations using, for instance, Vehicle-to-Anything (V2X) communications, can have a great impact on road safety.

ABIResearch [2] indicates that more than 10 million vehicles will be able to use short-range V2X communications by 2025 (if one considers cellular connectivity, that number may grow to 364 million). Naturally, achieving such a vast usage of V2X communication devices that are able to exchange important traffic data (information about the vehicle’s themselves, traffic conditions, etc.) results in huge amounts of data that are generated with high frequency.

Considering this, it seems pertinent to study whether this V2X data can be leveraged in the context of Machine Learning (ML). ML techniques enable the development of systems that have potential to improve traffic in general (both in terms of mobility and safety), e.g., predict road entity movement, probabilities of collisions, etc.

The implementation of such systems is (naturally) only possible if these users possess technologies that allow them to communicate with other generic road users. Even though some VRUs may have communication capabilities (e.g., pedestrians using their smartphone with cellular technologies), it is unlikely that they are able to communicate with other road agents in a direct way, as vehicles are typically equipped with different technologies (e.g., *IEEE 802.11p*). Nonetheless, regarding VRUs, motorcycles are fairly easy to equip with communication devices similar to regular vehicles (using similar On-Board Units (OBUs)). For this reason, motorcycles are the most appropriate subjects to use in the research of automatic solutions for VRU collision prediction utilizing ML applied to V2X communications data.

The proposed system is an extension of previous work conducted by the authors of [3], where a VRU accident *detection* system (focused on motorcycles) is presented. The system was built based on neuronal networks on top of simulated V2X communication data. Their results show that the system was able to detect all accidents between passenger vehicles and motorcycles on a intersection, averaging a detection time of 0.61 s. The detection of an accident in such a narrow time window may enable the trigger of *passive* safety mechanisms (e.g., calling an ambulance) or even some actions that aim to improve traffic mobility by minimizing the effects of the collision (e.g., notify surrounding vehicles that there is a collision on a nearby intersection). However, developing a system that is able to predict collisions allows for more active safety measures (e.g., automatic emergency breaking or notifying the driver of imminent danger). If such a prediction is achieved with enough antecedence (i.e., the road users have enough time to take action), it could greatly improve VRU safety.

This work presents a VRU (motorcyclist) collision prediction system, using ML technologies on V2X simulation data. The system proposes a solution to some of the aforementioned problems: the lack of safety solutions that try to leverage V2X data, as RADAR/camera systems do not work well in non-line-of-sight situations (unlike communications solutions), and the improvement of VRU safety by predicting collisions using (ML) time series forecasting techniques. Via simulation (using the VEINS framework with Simulation of Urban MObility (SUMO) and Network Simulator 3 (ns-3)) and subsequent data analysis, it is found that our system is suitable for practice.

This paper is organized as follows: Section 2 reviews the state of the art; Section 3 describes the systems architecture; Section 4 discusses the simulation scenario; Section 5 describes the process of training and testing the ML models; Section 6 discusses the main results of this work; Finally, Section 7 presents the main conclusions.

## 2. Related Work

Using ML solutions on V2X communications data can potentially improve traffic efficiency [4], both in terms of mobility (e.g., by predicting traffic flow [5,6]) and also the safety of the road users (e.g., by detecting and classifying road anomalies [7] and/or identifying crash risks [8]).

It is only natural and logical to try to apply similar techniques to VRU-focused safety. Although these users can easily change their trajectory [9,10], the application of ML techniques on environmental road data has the potential to predict their movement, classify their behavior or even predict the probability of collision.

Schneegans et al. [11] forecasted future trajectories of VRUs (cyclists) using two probabilistic trajectory forecasting methods: Quantile Surface Neural Networks and Mixture Density Neural Networks. The chosen use case was related to overtaking cyclists, and the authors relied on a trajectory dataset containing trajectories of cyclists crossing an intersection. Their results show that both methods issued well-calibrated and reliable confidence regions. Nevertheless, the Mixture Density Neural Network was able to issue smaller (and thus sharper) confidence regions, especially when considering higher forecasting horizons and larger coverage probabilities.

Dogru and Subasi [12] focused on finding a way to reduce the frequency and severity of traffic accidents by presenting an accident detection system that utilized V2V communications. Through this system, vehicles exchange their information (speed and coordinates) and send traffic alerts, which can be leveraged by machine learning techniques to detect accidents. Using SUMO to simulate traffic, the authors analyzed the Artificial Neural Network (ANN), Random Forest (RF) and Support Vector Machine (SVM) algorithms to evaluate their performance. Their results show that incidents can be regarded as outliers in data and, for this reason, machine learning techniques can be used to detect them, allowing later actuation, e.g., warning other vehicles about the incident. The RF algorithm was found to perform better when compared to the ANN and SVM.

Komol et al. [13] used real data from Queensland, Australia, from 2013 to 2019, to compare the performance of different machine learning algorithms when identifying crash severity factors for different VRUs: bicyclists, motorcyclists and pedestrians. The RF models performed best regarding the test accuracy (motorcyclist, 72.30%; bicyclist, 64.45%; pedestrian, 67.23%; unified VRU, 68.57%).

The work of Vilaça et al. [14] aimed to identify risk factors for VRUs that can affect their injury severity when involved in an accident. The model’s training involved the analysis of records related to VRU crash data. The results indicate that the Decision Tree (DT) method performed better than Logistic Regression (LR), as the model was more accurate for the available crash severity data. Nonetheless, both methods were able to correctly classify with relatively high accuracy.

Parada et al. [15] presented a VRU trajectory prediction system, using regression algorithms on Cartesian coordinates. When using a Alternating Model Tree, the system was able to predict the next position with negligible error (less than 3.2 cm). When predicting the next five positions (1 s time gap between consecutive positions), the error increased to 1 m.

Li et al. [16] proposed a machine learning method (a Support Vector Regression (SVR) model) for the prediction of lane-changing impacts on traffic. Records related to trajectory changes were obtained using the Next Generation Simulation (NGSIM) platform. Their results show that the models were able to reliably predict the lane change impacts on traffic safety and flow (based on the training and testing datasets). Furthermore, the authors concluded that motorcycles conducted lane changes with highest safety risks (smaller gaps and larger speed differences); trucks contributed to fewer but considerable crash risks (although their lane changes resulted in the largest flow reductions); regular automobiles were the safest vehicle type; and lane changes performed to the right had a more negative impact on the traffic flow and crash risk (when in comparison to lane changes to the left).

Most of the relevant related works on ML solutions applied to VRU safety tend to focus on data collected using sensors, cameras or similar devices, and not so much on data gathered via V2X communication.

Nonetheless, the implementation of collision prediction systems for VRUs utilizing ML (either using sensor or communications data) requires heavy computation and large amounts of storage for the training and testing of the models. Furthermore, the deployment and usage of the model/application also requires very low latencies to allow the users enough time to safely act on the predictions. As a safety application, it is critical that the prediction is achieved within a reasonable time, including the exchange of information and also the real-time analysis and treatment of such data.

A highly suitable solution for such a use case is utilizing the Fog Computing paradigm. The usage of Fog Computing introduces great benefits in terms of low latency and mobility, given that it performs tasks of computation, communication and storage near the edge of the network [17]. By using a distributed network of devices, this paradigm of computing (in comparison to more traditional Cloud architectures) brings applications and services from the Cloud to the edge of the network, greatly reducing the transfer times and meeting the demands of real-time applications (such as the short-term prediction of collisions).

Gomes et al. [18] presented an interesting and extensive survey on time-sensitive applications in fog computing environments, classifying the surveyed articles into five categories: the Fog Computing Concept, Faster Response, Low Latency, Data Streaming Application and the Time, Delay or Latency Constraint.

Liu et al. [19] presented a hierarchical system architecture using both software-defined networking and fog computing in IoV paradigms. The architecture consists of four layers: the application layer, the control layer, the virtualization layer and the data layer. The system was tested by implementing two real-world environment prototype services:

 **See Through**  This service aims to share a real-time view of a front vehicle to its following vehicles. The vehicle that intends to share its view registers at the SDN controller using LTE. Then, based on the vehicle topology (and registered services), the SDN controller notifies available services to particular vehicles via control messages. Vehicles are then able to request the services from the SDN controller using LTE. Once the service starts, the video can be streamed from the provider to the requesting vehicles using DSRC at the fog layer.
 **Collision Warning**  This service triggers warning messages when a potential collision between two vehicles is detected. The SDN controller communicates with vehicles via LTE. To support a large-scale and real-time service, the computation and communication workload is offloaded onto the fog server. The vehicle sends constantly up-to-date information using DSRC to the fog server (10 Hz), which then processes the data and estimates whether there is a risk of collision: in positive cases, the warning message is triggered and sent to the vehicles, which is then displayed on a HMI (along with sound and vibration).

Liu et al. [19] proposed an infrastructureless architecture (fog-based) named PV-Alert (Pedestrian–Vehicle Alert). In this architecture, the fog nodes process delay-sensitive data (that are obtained from smartphones) and generate alerts for pedestrians and drivers when an imminent collision is detected. The collected data are also sent to the Cloud for further analysis. The proposed solution was evaluated using the ns-3 and SUMO simulation tools (to simulate communications and mobility, respectively). The architecture was compared to other (smartphone) VRU-related safety architectures, and the results show that it scaled well and was reliable, while also providing low latencies.

## 3. Systems Architecture

As stated before, using Fog Computing brings many benefits in terms of latency and mobility, which are of utmost importance for safety applications in ITS environments. Furthermore, ML systems for ITSs may also benefit from the fact that it provides great capabilities of computation, communication and storage near the edge users of the network, while still meeting the tight latency requirements.

**Figure 1:**
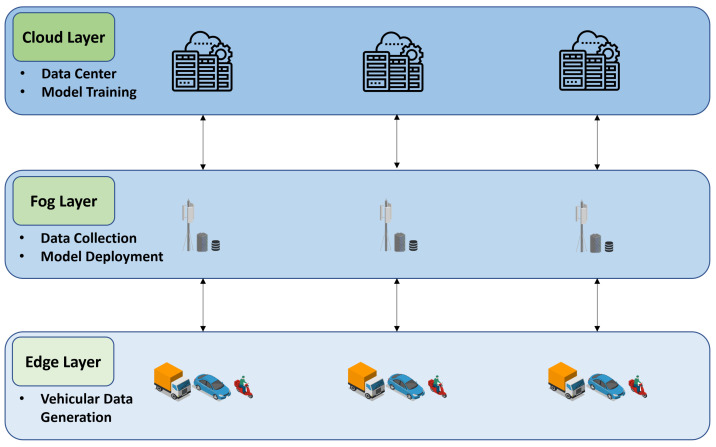
Systems hierarchical architecture.

Figure 1 shows the hierarchical architecture of the proposed system, following a Fog Computing paradigm.

As illustrated in the figure, the architecture is composed of three hierarchical layers:

 **Edge Layer**  This layer is the closest one to the end users (drivers/vehicles), which are typically widely distributed in geographical terms. Most of the regular vehicles that travel on the road are equipped with a large number of sensors. The information that is collected by such sensors can be shared with other entities on the road using OBUs with communication capabilities. This information may be useful to other users in this layer, particularly for Roadside Units (RSUs) on the Fog Layer.
 **Fog Layer**  This layer is situated on the edge of the core network. The nodes in this layer (Fog Nodes) are also widely distributed; for instance, they can be located in every intersection on the road. They are responsible for interconnecting the Cloud and the end users, which aim to obtain services from these nodes. Additionally, they have ample capability to perform heavier computation and to transmit/receive data (and also to process and store it). The implementation of low-latency and real-time applications can be achieved in this layer. Thus, this is the ideal location to deploy the models for the prediction of collisions: the application can receive the data that is disseminated from the surrounding vehicles, treat it (aggregate it) and use it for the prediction of collisions. The Fog Nodes also connect with the top layer (Cloud Layer) in order to obtain more powerful computing and storage capabilities.
 **Cloud Layer**  The Cloud Layer consists of servers and storage devices that possess great performance capabilities (powerful computation and ability to store huge amounts of data) and can provide several services. An example of such service is the prediction of collisions related to VRUs using ML application, using the ability to store the data that is sent from the Fog Nodes on the underlying layer and use it to (re)train the ML models.

**Figure 2:**
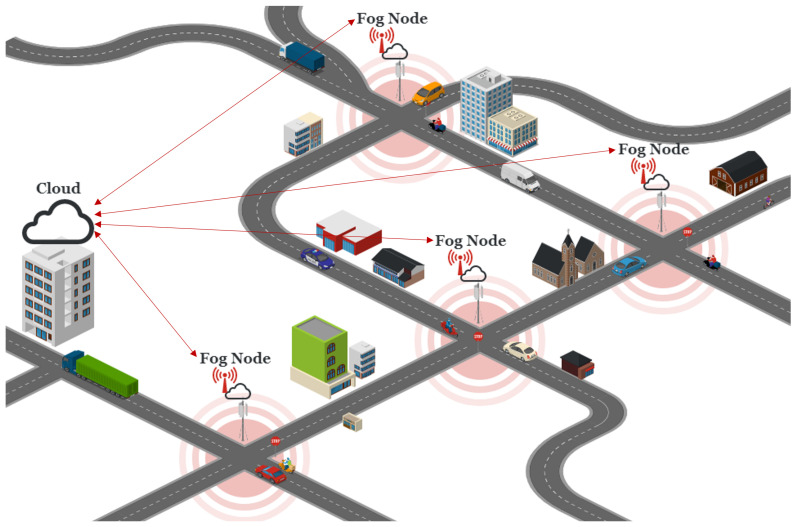
Systems architecture: the Prediction of collisions related to the VRU use case.

An example of the deployment of the complete system is illustrated in Figure 2.

In this architecture, each end device (vehicle) connects with a Fog Node, which is located at a road intersection, using wireless access technologies (e.g., IEEE 802.11p). The Fog Nodes can be connected to the Cloud using the IP core network.

The application of predicting collisions related to VRUs using ML can be divided into two stages:

 **Offline**  At the first offline stage, it is important to collect sufficient data to parametrize, train/test and deploy ML models, which will later be used for the prediction of the collisions. The end users (vehicles) broadcast data with fast rates (for instance, using Basic Safety Messages (BSMs) every 100 ms) that can be collected by the Fog Node, which is placed at an intersection (acting as a RSU). These data can then be treated and aggregated to be sent to the Cloud, which possesses better capabilities to store the huge amount of data and uses it to train the models, in this case, in a supervised manner. Thus, the Fog Node should also complement the collected data with information related to collision history, which is useful for the training process. When the process of training and testing the model is finished, the resulting weights and models can be sent back to the corresponding Fog Node in order to be deployed. This process should be repeated for each Fog Node.
 **Online**  When the first stage is accomplished, a model can finally be deployed on a Fog Node in order to start the prediction of the collisions. The Fog Node collects the broadcasted messages, treats them and uses the compilation of data as the input for the prediction of a possible collision. The resulting aggregated data are also sent to the Cloud in order to be stored and later used for retraining purposes, allowing the continuous tuning of the model. The weights resulting from the retraining process are sent back to the Fog Node in order to update the model. When a collision is predicted by a Fog Node, a message is disseminated to the relevant end users to allow them to make an appropriate decision.

## 4. Use Case Scenarios

In order to establish the scenario and its requirements, several use cases from European Telecommunications Standards Institute (ETSI) standards were analyzed, such as Collision Risk Warning from RSU from [20], Turning Collision Risk Warning from [21] and, with particular emphasis, the Scooter/Bicyclist Safety with Turning Vehicle standard from [22].

**Figure 3:**
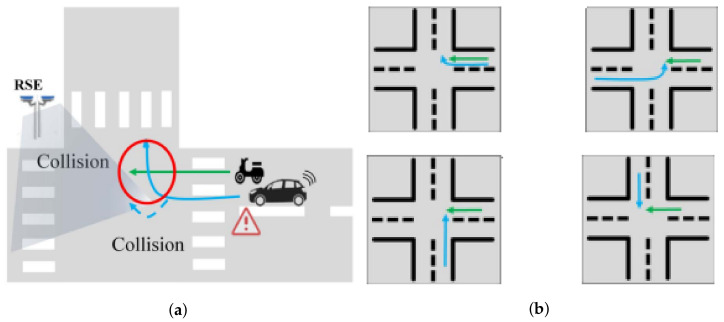
(**a**) Scooter/Bicyclist Safety with Turning Vehicle Use Case; (**b**) Possible collision situations [22].

This use case, illustrated in Figure 3, is described as a critical traffic situation, where a vehicle makes a turn at an intersection and sees a scooter, which makes a collision between the agents possible (the figure also presents alternative collision situations).

In this use case, there is a RSU present at the intersection that is equipped with both a communication device and some form of sensor. This sensor is used to detect both the VRUs and the vehicles, and the information is used to predict their path and compute possible collisions. If an imminent collision is detected, the RSU broadcasts warning messages to the vehicles in the area. The vehicle, upon receiving the collision avoidance message, takes appropriate actions to avoid the accident.

In this example, the information that is used to feed the prediction mechanisms is collected via sensors (e.g., camera). However, and as discussed before, this type of solution may eventually perform poorly in situations where line of sight is non-existent or limited. For this reason, we intend to study the feasibility of utilizing vehicular communications to feed such prediction mechanisms. The proposed use case is described below:

 **Description**  A passenger vehicle makes a turn at an intersection and sees an approaching motorcycle that intends to go straight on the road, which results in a possible collision.
 **Actors**  In this scenario, there are the following actors:
   **1. Passenger Vehicle**     Equipped with an OBU;
   **2. Motorcycle**              Equipped with an OBU;
   **3. RSU**                      Equipped with IEEE 802.11p and automatic mechanisms for the prediction of possible collisions, using ML.
 **Pre-conditions**  Passenger vehicles, motorcycles and one RSU are able to receive and broadcast standard vehicular messages. The RSU vehicle possesses mechanisms for the automatic prediction of possible collisions between passenger vehicles and motorcycles.
 **Triggers**  Motorcycle and passenger vehicle are close to the intersection; the motorcycle goes straight through the intersection; vehicles makes a turn at the intersection, crossing roads with the motorcycle’s straight route.
 **Normal Flow**  
  1. Passenger vehicles and motorcycles broadcast information using standard vehicular messages;
  2. The RSU receives the standard vehicular messages and collects the data from those agents;
  3. The RSU performs collision prediction using automatic mechanisms;
  4. The RSU broadcasts warning messages to vehicles in the area;
  5. The vehicles receive the collision avoidance message.
 **Post-conditions**  Vehicles are alerted of potential collisions and take appropriate actions to avoid or minimize the effects of collisions.

### Scenario Simulation Implementation

Two scenarios, based on two use cases from Figure 3b (top right and bottom left), were implemented in the simulation using the VEINS framework (which couples SUMO and OMNeT++): the other models could not be implemented due to the limitations of the SUMO simulator regarding collision behavior.

Concerning communications, all elements are communicating using IEEE 802.11p technology (WAVE/DSRC stack), exchanging BSM-like beacons with a 10 Hz frequency containing the following information:

- Station ID;
- Longitude;
- Latitude;
- Altitude;
- Heading;
- Speed;
- Acceleration;
- Vehicle Length;
- Vehicle Width;
- Vehicle Type.

The default (out of the box) communication parameters for the network interface cards were used in the simulator. These parameters are presented in Table 1.

These messages are gathered by a RSU for 24 h of simulation and compiled into a dataset: each simulation run (and seed) results in a different dataset. Fifty simulation runs were used to compile the final dataset, which was later used by the models—80% for training, 10% for validation and 10% for testing. The resulting datasets are available at https://zenodo.org/record/7376770 [23] (accessed on 1 December 2022).

## 5. Collision Prediction System

The proposed use case is highly characteristic in terms of temporal concerns. This issue makes traditional ML models unsuitable to solve the problem, due to the sequence dependence among the input variables and the fact that they do not consider the time aspect at all. This was noticeable in the first iteration, in which several common ML prediction models were chosen for initial training and testing: Logistic Regression, K-Nearest Neighbors, Gaussian Naive Bayes, Support Vector Machines and Artificial Neural Networks. The algorithms were used to determine whether the records belonged to a collision or not, all achieving accuracy values above 80%. Although, at a first glance, the high values of the metrics may sound promising, the solutions were in fact very poor. Since there are only a few collisions occurring during the simulation (they are rare events), only a few thousand messages are effectively labeled as true (in a universe of several hundreds of thousand of messages). For this reason, the dataset is considered to be *imbalanced*: too many messages labeled as *false* when in comparison to the ones labeled as *true*. This helps to explain why the algorithms have high levels of accuracy at this point: even if the models classify every single record as *false*, the accuracy is very high, because only a small subset of the records is being classified inappropriately. Regarding the collisions classification itself (the problem that is to be solved), all models performed badly and were not able to classify properly. As the traditional models performed far worse than expected, a different technical approach is required in order to solve the problem, namely, time series forecasting.

The core idea of time series forecasting modeling is to examine data from a time perspective, defining patterns and predicting (over the short- or long-term) how target variables will change in the future. In other words, time series forecasting is a process of using historical and current data to predict future values (over a period of time or at a specific point in the future).

A highly suitable deep learning model for this type of use case is Long Short-Term Memory (LSTM). LSTM models have the capability to hold information for long periods of time: information that is learned early on can still impact the model’s decision later. The key idea of a Long Short-Term Memory (LSTM) cell is build upon the usage of three gates (weighted functions that govern the information flow and state):

 **Input Gate**  Takes input and processes newly incoming data (updates the internal state based on the current input).
 **Forget Gate**  Decides what information to discard from the internal state.
 **Output Gate**  Takes all calculated results and decides what to output based on the input and internal state (i.e., which information is passed on to the next state).

In order to implement a term of comparison, the usage of Multilayer Perceptrons (MLPs) was also tested, since they tend to perform well with tabular datasets and in classification problems where the inputs are assigned a label (in this case, *collision* or *not in collision*). However, and after several runs of training and testing, one could conclude that the MLPs performances were poorer and, for this reason, the results ended up not being included in this discussion.

As stated before, another key aspect of the proposed scenario is that collisions are rare events: data are *imbalanced*. First, in order to try to overcome this issue, different class weights were estimated—the model’s loss function was assigned a higher value compared to the positive instances, which are rarer. However, later on, a different solution was tested that undersampled the negative cases. This method outperformed the first, and was also very useful due to the fact that the collection of training data was extensive, which made the learning process slow and heavy in terms of computation. Hence, data were truncated on those large periods of time where no collisions occurred. Different time windows (750 s, 1000 s, 1500 s, 2000 s and 2500 s) were tested, e.g., keep 2000 s before and after every collisions, while removing the remaining information.

Furthermore, the datasets consist of (singular) messages that the vehicles were disseminating (through the use of communications), collected by a RSU at an intersection. However, performing the classification requires the model to be aware of changes in the whole environment in order to make informed decisions. Therefore, to solve the problem of possessing a large collection of *singular* data, the messages were aggregated in a temporal fashion: all messages within 1 s were compressed into a single record. Different methods were tested for this aggregation (min, max, sum and average), but the sum method outperformed the others. When comprising the information, Station ID, Vehicle Type and Timestamp were removed as features, as they did not make sense when aggregated. Elevation was also removed, as every value was equal to zero in this particular simulation scenario; hence, it did not make sense to use it in the model. Finally, a new feature was added—Vehicle Count—which indicates how many vehicles sent messages during that particular second.

**Figure 4:**
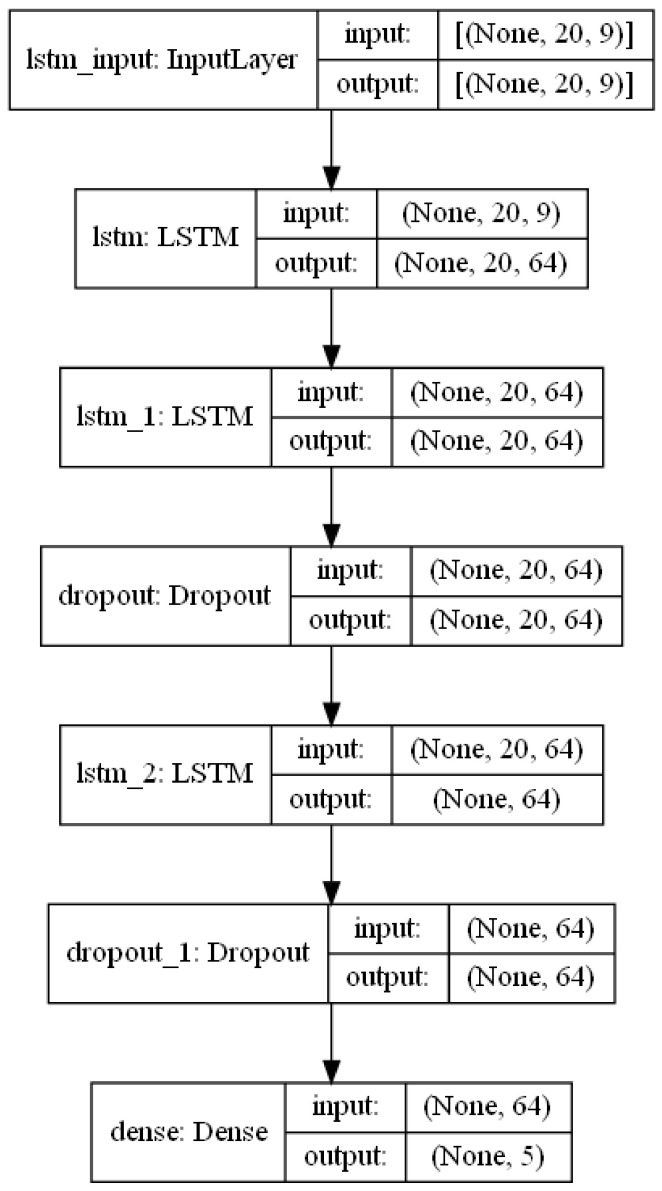
LSTM model summary—*A6 Run* (five multisteps).

Different variations of LSTM models were explored: first, a model with one LSTM layer was tested, then a model with two unidirectional LSTM layers, then two unidirectional LSTM layers with dropout (to prevent overfitting), and similar configurations. The solution that had the best performance consisted of a multivariate, multistep, stacked, unidirectional model that used three hidden LSTM layers and two dropout layers, with all features being used in the input layer. A summary of the models is illustrated in Figure 4 (using, as an example, the *A6 Run* with five steps).

Table 2 presents the remaining hyperparameters used to build the models. In this case, no optimization method was used. The usage of *Grid Search* was very costly in computational terms (which made it unfeasible) and *Random Search* eventually resulted in similar results in terms of typical metrics (e.g., accuracy, precision, recall), but worse results in terms of the accident prediction in the analysis after the training and testing processes.

The models were developed using the *tensorflow* framework [24] (version tensorflow-gpu 2.4.1). A Python script was developed to perform all of the aforementioned steps.

The pseudocode is illustrated as follows:


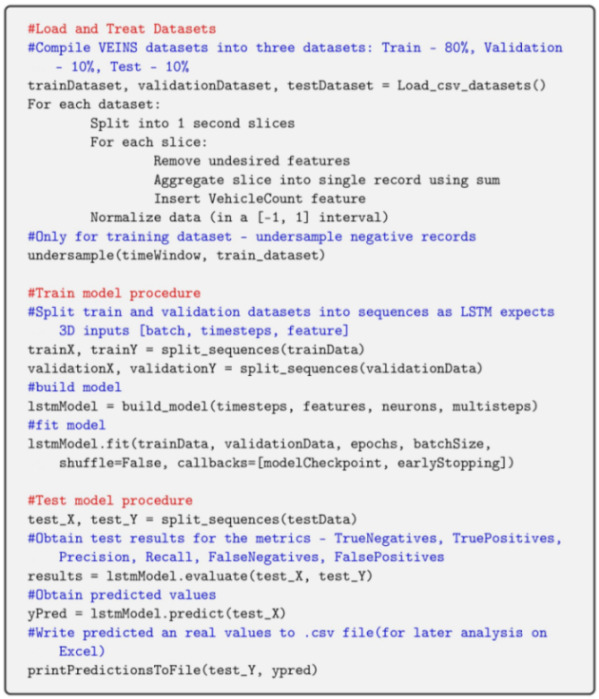


Regarding the model fitting process (which went up to a maximum of 1000 epochs), two different callbacks were defined:

 **ModelCheckpoint**  Used to save the weights of the model into a checkpoint file. This is useful so the weights can be loaded later to continue the training from the saved state.
 **EarlyStopping**  Useful for stopping the training when a monitored metric is no longer improving. In this case, the callback monitors the *validation loss* metric, with a patience of 20 epochs for improvement.

## 6. Results

This section describes the results from the training and testing of the models, which were performed in an iterative fashion. First, different sets of parameters (Time Windows, Batch Size, Neurons and Timesteps) were tested for *one-step ahead forecast*, which, in this case, means forecasting one second into the future. Then, only the best set of parameters (Correct Decision Percentage (CDP) higher than 33%; this metric is further discussed below) were then used to perform *multistep forecasting*. This approach has the disadvantage of possibly not presenting optimal results. It is possible that some other set of parameters could eventually perform better. This decision was related to practical reasons: performing training and testing on all sets of possible parameters is highly consuming, both in terms of time and computation.

Table 3 presents the best results achieved for both scenarios regarding the one-step ahead forecast.

The first part on the table (on the left side) presents the set of parameters used on the corresponding run. Then, the middle section presents some partial results of the *Model.evaluate()* function (available on the *Tensorflow* built-in API), which returns both the loss and metric values of the model after testing (using the test dataset).

In this use case, it is not possible to simply decide which models perform best based on typical metrics such as Accuracy or Precision, Recall or F-Beta: all values were very close to 1 (100%). Hence, a more in-depth analysis was performed using Microsoft Excel (results visible on the right section of the table). The most important metrics are, in the first instance, the Collision Prediction Percentage (CPP) (number of collisions from the test dataset that were in fact predicted) and also the number of False Positives (FPs) (situations where the model wrongly forecasts a collision, which is an undesired situation). In order to make a proper decision related to these two metrics, a new metric was defined:

$$CDP=\frac{\mathrm{Predicted}\mathrm{Collisions}}{\mathrm{FPs}+\mathrm{Total}\mathrm{Collisions}}$$

This metric gives a value related to the number of correct decisions on the critical points of decision: the collisions themselves and the FPs instances. Taking, as an example, Scenario A, one can consider that the best-performing run (regarding CDP) is *A3*, with 55% correct decisions. Although, for instance, *A6* predicted more accidents correctly (44 vs. 42), the number of FPs is also much higher (36 vs. 20), which ultimately leads to a worse performance.

Hence, the choice of the best-performing model is mostly based on CDP, which relates both metrics, and is thus not simply based on the number of total predicted accidents.

As stated before, these best-performing run parameters were then used to train and test new models for the Multi-steps (MS) forecasting, of which the results are presented in Table 4.

The results are now organized and discussed by multistep groups. Table 5 presents the results for the two MS runs, for both Scenario A and B.

In Scenario A, the two best-performing models were from run *A2* and run *A3* (only models with a CDP over 50%). One can consider that run *A2* outperforms run *A3*: it predicted more accidents (91% vs. 88%), and despite having an higher FPs value (43 vs. 42), it still had a better CDP final value (52% vs. 51%). Thus, run *A2* is the best-performing model when considering two MS forecasting.

In Scenario B, the higher CDP values were achieved by run *B2* and run *B6*, with 47% and 46%, respectively. However, in this case, and despite the fact that the first one achieved an higher value for CDP, its Accident Prediction Percentage is much lower (72% vs. 81%). Considering that the Average Prediction Time is similar for both, run *B6* can be considered the best-performing model.

Table 6 illustrates the results for the three MS forecasts (Scenario A and B).

In Scenario A, the results in terms of CDP are very similar between the runs (except for run A1, with the lowest CDP value of 42%). In terms of CPP, runs *A3, A4* and *A7* performed equally well with 91%. These runs differ only in terms of FPs and the Average Prediction Time (s) (APT). Run *A4* had the best APT (2.90s) (runs *A3* and *A7* were very similar, with 2.67 s and 2.65 s). In terms of FPs, run *A3* had the lowest number—40 vs. 45 and 41. Thus, taking all values into consideration, run *A3* was considered the best-performing overall.

In Scenario B, the CDP values became globally higher when comparing to the two MS results. Here, the two models with highest CDP were run *B5* and run *B6*. Both performed similar in terms of FPs (41 vs. 42) and APT (3 s vs. 2.87 s). The main difference relies on the CPP, for which run *B6* had the higher value, 95% vs. 88%, than run *B5*. Hence, run *B6* is considered to have the best-performing model.

Table 7 shows the results for the four MS runs in both scenarios.

In Scenario A, only run *A4* achieved a CDP greater than 50%. Despite having other runs with better CPP values (e.g., runs *A3, A5* and *A6*), they also had more FPs. Since run *A4* was the only run that could at least make a correct decision in every two (>50%), it is considered the best-performing run.

In Scenario B (similarly to Scenario A), only one run (*B6*) was able to achieve a CDP over 50–57%. Runs *B4, B5* and *B7* had similar results for APP, but the number of FPs was much higher, which made them less suitable solutions.

Finally, Table 8 presents the results for the five MS forecast.

In Scenario A, not a single model was able to achieve a CDP over 50%. Despite having excellent results in terms of CPP (all above 90%), they all also had a high number of FP classifications, which caused lower CDP values. Run *A1* had fewer FPs, but, on the other hand, it also had the lowest APT. Considering all metrics, run *A4* had the best performance, with the highest values for CPP and CDP.

Similarly, in Scenario B, none of the models were able to achieve a CDP greater than 50%, and they all had invalid results for CPP (the lowest value was still a prediction of 50 out of 57). Again, all the models presented relatively high numbers of FPs. Run *B4* can be considered the best: it had fewer FPs and an APT of 4.44 s (most of the runs also performed at around 4.5 s, except run *2*).

### Summary

This subsection discusses the best obtained results, which are summarized in Table 9.

When considering the best-performing runs, the system globally achieved very good results in terms of CPP, as most collisions were predicted correctly—the worst performance was achieved in 2 MS for Scenario B, where still 81% of the accidents were predicted. Although the models performed well for CPP, the main drawback comes from the high number of FPs. This higher value also ultimately resulted in lower values for the CDP—roughly one in every two critical decisions made by the system were correct.

When comparing the usage of two or three MS, it is possible to conclude that it is better to utilize the latter: it achieved better CPPs and CDPs. Furthermore, the models also performed better in terms of APTs—the higher values means that the collisions were also predicted sooner.

Between three and four MS, the results are very similar in terms of CDP and APT. However, on the four MS case, the CPP values are lower. Hence, no performance gain is noted when increasing the MS for the forecast. In this case, using extra computation for a longer forecast does not pay off.

Finally, regarding the maximum value for MS (five), the CPP values are very good (both above 95%), but the number of FPs are very high, which leads to lower values for CDP. Despite making less accurate decisions, the APT values are much higher (around 4.5 s in both cases).

All things considered, our results can be looked at from two perspectives: if one considers that CPP and CDP are a priority, using three MS is the best solution; however, using five MS is better if higher APT are preferred (despite having lower CDP values)—higher APT gives drivers more time to receive the warning notification, analyze the environment and take action if necessary. However, one must be conscious that using five MS has the drawback of having slightly lower CDP: roughly four in every ten predictions result in an actual collision (a high number of warning notifications received by the drivers are in fact false alarms). On the other hand, the system may be able to avoid (at least) 95% of the accidents if the drivers are able to manually act in those 4.5 s (roughly). Hence, a trade-off is also noted here.

Nevertheless, an explanation for the high number of FPs is related to the scenario’s implementation on the SUMO simulator. SUMO does not have collision simulation by default, and they had to be deliberately caused by parameterization. Hence, there are *near-accident* incidents happening throughout the simulation runs. On such occasions, the mobility patterns and configurations of the vehicles are very similar to those for accidents; however, the road users do not actually end up colliding.

**Figure 5:**
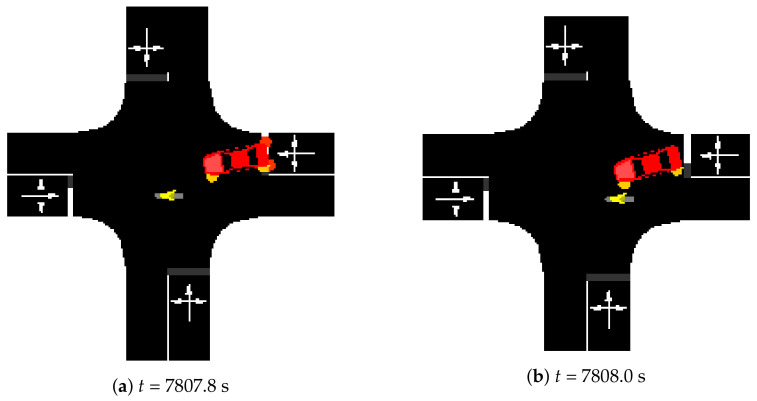

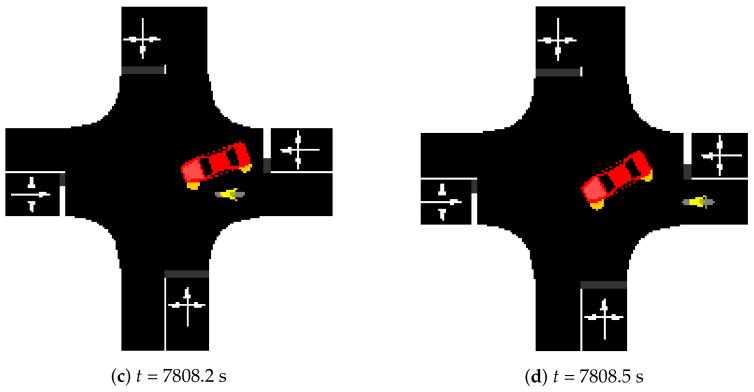
*Near-collision* example: Scenario A, *seed* 0.

Figure 5 illustrates an example of a *near-collision* incident (from Scenario A—*seed* 0).

The picture illustrates an example of an incident where a car turning left and a motorcycle going straight almost end up colliding. From the model’s point of view, this situation is very similar to the simulated accidents, which leads to incorrect positive predictions. Naturally, from a purely statistical view, these cases are still counted as FPs. Unfortunately, counting these particular cases is very hard to achieve: it must be performed manually while looking at the simulator running close to real time, which makes this analysis impractical.

Considering this, not all FP classifications mean necessarily bad results: a notification of imminent danger that is related to these *near-collision* incidents may also help to avoid them, since the drivers have enough time to adjust their behavior and proceed more carefully (also helping to increase safety on the roads).

## 7. Conclusions and Future Work

This work describes the development and testing of a system that aims to improve road safety for Vulnerable Road Users (VRUs) (motorcyclists) by predicting collisions through means of Machine Learning (ML).

In order to build datasets to feed the proposed stacked, unidirectional Long Short-Term Memory (LSTM) models, two different simulation scenarios containing collisions between passenger vehicles and motorcycles at an intersection were developed using VEINS (which couples Simulation of Urban MObility (SUMO) and OMNeT++).

From the obtained results, the system performs best when using five Multi-steps (MS) (5 s into the future) for the prediction (although the results present a lower Correct Decision Percentage (CDP) in comparison to fewer MS).

In Scenario A, 96% of the collisions were predicted with a 4.53 s average and a CDP of 41% (with a total of 78 False Positives (FPs)); In Scenario B, 95% of the collisions were predicted with a 4.44 s average, while the CDP was 43% (68 FPs).

The proposed system allows the usage of safety measures that could greatly improve VRU safety on the roads. However, due to the relatively high number of False Positives, it does not allow the implementation of *automatic* safety mechanisms (e.g., automatic emergency breaking), since is not desirable to actuate in such cases. Hence, the best way of actuating is *passively*, by warning the drivers of the vehicles of imminent collision, leaving to them the performance of defensive and preventive actions. Nonetheless, some FP cases were related to *near-collision* incidents that occur throughout the simulation runs, where the mobility patterns of the involved vehicles were very similar to those of regular collisions. Since these situations may also be avoided (although they are counted as FPs from a purely statistical view), not all FP cases should necessarily be seen as bad results.

Although the system performed well for two different scenarios (with consistent results), the data used to test it were generated and collected by means of simulation. Naturally, such solution limits the realism and applicability of the solution in the real world, which is the main limitation of this work.

At this point, the authors are working on deploying the model on the simulation platform, utilizing the Python-C API, and connecting the VEINS framework application (*C++*) to the Python script, in order to allow the treatment of the collected data and also predict probabilities of collisions in the next steps as the simulation is running.

As future work, the authors also intend to test the implementation of safety measures on the simulation framework to study their effects on traffic, especially in terms of safety, but also on mobility.

## Figures and Tables

**Table 1:** OMNeT++ Network Interface Card, 802.11p, specific parameters.

Network Interface Card Parameters	
Tx Power	20 mW
Bit Rate	6 Mbps
Min Power Level	−110 dBm
Noise Floor	−98 dBm
Decider	
*Decider80211p*	Center frequency = 5.89 GHz
Analogue Model	
*SimplePathlossModel*	α = 2.0

**Table 2:** LSTM model hyperparameters.

Model Hyperparameters	
LSTM Layers Activation Function	tanh
Dense Layer (Output) Activation Function	sigmoid
Dropout Layers	rate = 0.3
Optimizer	adam
Loss function	binary_crossentropy

**Table 3:** One-Step Ahead Forecast Results, Part I. Legend: TW—Time Window; BS—Batch Size; N—Neurons; TS—Timesteps; PC—Predicted Collisions; CPP—Collision Prediction Percentage; FP—False Positives; CDP—Correct Decision Percentage.

Run	TW	BS	N	TS	Precision	Recall	F-Beta	PC	CPP	FP	CDP
**Scenario A**											
A1	2000	256	10	10	0.9981	0.9990	0.9985	38	67%	39	40%
A2	2000	128	32	10	0.9979	0.9993	0.9986	41	72%	27	49%
A3	1500	256	32	15	0.9986	0.9990	0.9988	42	74%	20	55%
A4	1500	128	32	20	0.9986	0.9825	0.9905	40	70%	30	46%
A5	2000	128	64	15	0.9977	0.9983	0.9980	41	72%	36	44%
A6	1500	256	64	20	0.9979	0.9992	0.9986	44	77%	36	47%
A7	2000	256	64	10	0.9981	0.9991	0.9986	37	65%	22	47%
**Scenario B**											
B1	1500	128	32	10	0.9981	0.9993	0.9987	41	72%	52	38%
B2	2000	128	32	15	0.9982	0.9994	0.9988	41	72%	34	45%
B3	2000	128	32	20	0.9978	0.9991	0.9984	43	75%	42	43%
B4	1500	128	64	15	0.9981	0.9992	0.9987	35	61%	27	42%
B5	1500	256	64	20	0.9984	0.9990	0.9987	36	63%	18	48%
B6	2000	256	64	20	0.9985	0.9990	0.9988	37	65%	15	51%
B7	1500	256	64	10	0.9978	0.9992	0.9985	44	77%	54	40%

**Table 4:** MS Forecast Results. Legend: MS—Multiple steps; FPs—False Positives; PC—Predicted Collisions; CPP—Collision Prediction Percentage; CDP—Correct Decision Percentage; APT—Average Prediction Time (s).

Scenario A							Scenario B						
**Run**	**MS**	**FPs**	**PC**	**CPP**	**CDP**	**APT (s)**	**Run**	**MS**	**FPs**	**PC**	**CPP**	**CDP**	**APT (s)**
A1	2	51	51	89%	47%	1.76	B1	2	73	42	74%	32%	2.00
	3	54	47	82%	42%	2.49		3	51	50	88%	46%	2.88
	4	44	49	86%	49%	2.29		4	57	51	89%	45%	3.00
	5	69	52	91%	41%	3.33		5	96	54	95%	35%	4.59
A2	2	43	52	91%	52%	1.81	B2	2	31	41	72%	47%	2.00
	3	34	47	82%	52%	2.70		3	58	41	72%	36%	3.00
	4	55	50	88%	45%	2.48		4	36	35	61%	38%	2.60
	5	81	55	96%	40%	4.51		5	86	50	88%	35%	4.04
A3	2	42	50	88%	51%	2.06	B3	2	49	36	63%	34%	1.97
	3	40	52	91%	54%	2.67		3	57	42	74%	37%	2.57
	4	59	53	93%	46%	2.74		4	68	48	84%	38%	2.83
	5	170	54	95%	24%	4.56		5	89	54	95%	37%	4.59
A4	2	45	43	75%	42%	1.98	B4	2	53	43	75%	39%	2.02
	3	45	52	91%	51%	2.90		3	57	48	84%	42%	2.77
	4	39	49	86%	51%	2.76		4	47	51	89%	49%	2.90
	5	78	55	96%	41%	4.53		5	68	54	95%	43%	4.44
A5	2	39	45	79%	47%	1.82	B5	2	61	50	88%	42%	1.90
	3	39	49	86%	51%	2.76		3	41	50	88%	51%	3.00
	4	49	52	91%	49%	2.88		4	55	52	91%	46%	2.90
	5	84	53	93%	38%	4.26		5	73	54	95%	42%	4.48
A6	2	49	47	82%	44%	2.00	B6	2	43	46	81%	46%	1.98
	3	36	49	86%	53%	2.63		3	42	54	95%	55%	2.87
	4	64	54	95%	45%	2.72		4	33	51	89%	57%	2.94
	5	82	55	96%	40%	4.56		5	80	53	93%	39%	4.45
A7	2	38	47	82%	49%	2.00	B7	2	53	47	82%	43%	1.98
	3	41	52	91%	53%	2.65		3	75	53	93%	40%	2.75
	4	49	50	88%	47%	3.02		4	58	52	91%	45%	2.94
	5	88	54	95%	37%	4.50		5	105	54	95%	33%	4.54

**Table 5:** Two MS results. Legend: R—Run; FPs—False Positives; PC—Predicted Collisions; CPP—Collision Prediction Percentage; CDP—Correct Decision Percentage; APT—Average Prediction Time (s).

R	FPs	PC	CPP	CDP	APT	R	FPs	PC	CPP	CDP	APT
**Scenario A**						**Scenario B**					
A1	51	51	89%	47%	1.76	B1	73	42	74%	32%	2.00
A2	43	52	91%	52%	1.81	B2	31	41	72%	47%	2.00
A3	42	50	88%	51%	2.06	B3	49	36	63%	34%	1.97
A4	45	43	75%	42%	1.98	B4	53	43	75%	39%	2.02
A5	39	45	79%	47%	1.82	B5	61	50	88%	42%	1.90
A6	49	47	82%	44%	2.00	B6	43	46	81%	46%	1.98
A7	38	47	82%	49%	2.00	B7	53	47	82%	43%	1.98

**Table 6:** Three MS results. Legend: R—Run; FPs—False Positives; PC—Predicted Collisions; CPP—Collision Prediction Percentage; CDP—Correct Decision Percentage; APT—Average Prediction Time (s).

R	FPs	PC	CPP	CDP	APT	R	FPs	PC	CPP	CDP	APT
**Scenario A**						**Scenario B**					
A1	54	47	82%	42%	2.49	B1	51	50	88%	46%	2.88
A2	34	47	82%	52%	2.70	B2	58	41	72%	36%	3.00
A3	40	52	91%	54%	2.67	B3	57	42	74%	37%	2.57
A4	45	52	91%	51%	2.90	B4	57	48	84%	42%	2.77
A5	39	49	86%	51%	2.76	B5	41	50	88%	51%	3.00
A6	36	49	86%	53%	2.63	B6	42	54	95%	55%	2.87
A7	41	52	91%	53%	2.65	B7	75	53	93%	40%	2.75

**Table 7:** Four MS results. Legend: R—Run; FPs—False Positives; PC—Predicted Collisions; CPP—Collision Prediction Percentage; CDP—Correct Decision Percentage; APT—Average Prediction Time (s).

R	FPs	PC	CPP	CDP	APT	R	FPs	PC	CPP	CDP	APT
**Scenario A**						**Scenario B**					
A1	44	49	86%	49%	2.29	B1	57	51	89%	45%	3.00
A2	55	50	88%	45%	2.48	B2	36	35	61%	38%	2.60
A3	59	53	93%	46%	2.74	B3	68	48	84%	38%	2.83
A4	39	49	86%	51%	2.76	B4	47	51	89%	49%	2.90
A5	49	52	91%	49%	2.88	B5	55	52	91%	46%	2.90
A6	64	54	95%	45%	2.72	B6	33	51	89%	57%	2.94
A7	49	50	88%	47%	3.02	B7	58	52	91%	45%	2.94

**Table 8:** Five MS results. Legend: R—Run; FPs—False Positives; PC—Predicted Collisions; CPP—Collision Prediction Percentage; CDP—Correct Decision Percentage; APT—Average Prediction Time (s).

R	FPs	PC	CPP	CDP	APT	R	FPs	PC	CPP	CDP	APT
**Scenario A**						**Scenario B**					
A1	69	52	91%	41%	3.33	B1	96	54	95%	35%	4.59
A2	81	55	96%	40%	4.51	B2	86	50	88%	35%	4.04
A3	170	54	95%	24%	4.56	B3	89	54	95%	37%	4.59
A4	78	55	96%	41%	4.53	B4	68	54	95%	43%	4.44
A5	84	53	93%	38%	4.26	B5	73	54	95%	42%	4.48
A6	82	55	96%	40%	4.56	B6	80	53	93%	39%	4.45
A7	88	54	95%	37%	4.50	B7	105	54	95%	33%	4.54

**Table 9:** Collision prediction summary results. Legend: MS—Multiple steps; FPs—False Positives; PC—Predicted Collisions; CPP—Collision Prediction Percentage; CDP—Correct Decision Percentage; APT—Average Prediction Time (s).

MS	Scenario	Run	FPs	PC	CPP	CDP	APT (s)
2	A	A2	43	52	91%	52%	1.81
	B	B6	43	46	81%	46%	1.98
3	A	A3	40	52	91%	54%	2.67
	B	B6	42	54	95%	55%	2.87
4	A	A4	39	49	86%	51%	2.76
	B	B6	33	51	89%	57%	2.94
5	A	A4	78	55	96%	41%	4.53
	B	B4	68	54	95%	43%	4.44

## Data Availability

The datasets presented in this study are available in Zenodo at https://doi.org/10.5281/zenodo.7376770 (accessed on 16 December 2022), reference number [23]. These datasets are the raw data used for the testing and training of the ML algorithms in this work.

## Abbreviations

The following abbreviations are used in this manuscript:

ANN	Artificial Neural Networks
APT	Average Prediction Time (s)
BS	Batch Size
BSM	Basic Safety Messages
CDP	Correct Decision Percentage
CPP	Collision Prediction Percentage
ETSI	European Telecommunications Standards Institute
FPs	False Positives
ITS	Intelligent Transportation Systems
LSTM	Long Short-Term Memory
ML	Machine Learning
MLP	Multilayer Perceptron
MS	Multi-steps
N	Neurons
OBU	On-Board Unit
PC	Predicted Collisions
RSU	Roadside Unit
SUMO	Simulation of Urban Mobility
TS	Timesteps
TW	Time Window
V2X	Vehicle-to-Anything
VRU	Vulnerable Road User
